# The risk prediction of intergenerational transmission of overweight and obesity between mothers and infants during pregnancy

**DOI:** 10.1186/s12884-024-06268-7

**Published:** 2024-01-23

**Authors:** Xiaotong Wei, Jiajin Hu, Deliang Wen

**Affiliations:** 1 Institute of International Health Professions Education and Research, China Medical University, Shenyang, 110122 Liaoning Province China; 2 Institute of Health Sciences, China Medical University, Shenyang, 110122 Liaoning Province China

**Keywords:** Prediction model, Intergenerational transmission, Maternal overweight, Maternal obesity, Infant overweight, Infant obesity

## Abstract

**Background:**

Overweight and obesity in mothers before pregnancy lead to overweight and obesity in their offspring, which is the main form of intergenerational transmission of overweight and obesity in early life. Many factors, especially non-genetic factors, may influence intergenerational transmission, but little prediction research has been conducted. Therefore, we analyzed the status of intergenerational transmission in maternal and infant overweight and obesity. Second, we explored the factors during the pregnancy that might affect the the intergenerational transmission; According to the two application scenarios of pregnancy screen and self-management, risk prediction models for pregnant women were carried out.

**Methods:**

Based on a prospective birth cohort, a total of 908 mothers and offspring were followed up during early life. Follow-up visits were performed at the first trimester, second trimester, third trimester, delivery, 42 days after delivery, and 6 months and 12 months of age. The investigation methods included questionnaire survey, physical examination, biological sample collection and clinical data collection. In terms of risk prediction, univariate analysis was used to screen candidate predictors. Second, multivariable Cox proportional hazard regression models were used to determine the final selected predictors. Third, the corresponding histogram models were drawn, and then the 10-fold cross-validation methods were used for internal verification.

**Results:**

Regarding intergenerational transmission of overweight and obesity between mothers and infants during pregnancy, the risk prediction model for pregnancy screen was constructed. The model established: h(t|X) = h_0_(t)exp.(− 0.95 × (Bachelor Degree or above) + 0.75 × (Fasting blood glucose in the second trimester) + 0.89 × (Blood pressure in the third trimester) + 0.80 × (Cholesterol in third trimester) + 0.55 × (Abdominal circumference in third trimester))., with good discrimination (AUC = 0.82) and calibration (Hosmer–Lemeshow^2^ = 4.17). The risk prediction model for self-management was constructed. The model established: h(t|X) = h_0_(t)exp. (0.98 × (Sedentary >18METs) + 0.88 × (Sleep index≥8) + 0.81 × (Unhealthy eating patterns Q3/Q4) + 0.90 × (Unhealthy eating patterns Q4/Q4) + 0.85 × (Depression)), with good discrimination (AUC = 0.75) and calibration (Hosmer–Lemeshow^2^ = 3.81).

**Conclusions:**

The risk predictions of intergenerational transmission of overweight and obesity between mothers and infants were performed for two populations and two application scenarios (pregnancy screening and home self-management). Further research needs to focus on infants and long-term risk prediction models.

**Supplementary Information:**

The online version contains supplementary material available at 10.1186/s12884-024-06268-7.

## Background

Children with obesity has become a global public health challenge. Recent studies suggest that children with obesity might be the result of intergenerational transmission [1]. Intergenerational transmission is defined as “the conditions, exposures, and environmental factors experienced by one generation that are relevant to the health, growth and development of the next generation [2].” Overweight and obesity in mothers before pregnancy lead to overweight and obesity in their offspring, which is the main form of intergenerational transmission of overweight and obesity in early life [3]. Mothers who are overweight and obese before pregnancy expose the fetus to an adverse metabolic environment in the womb, increasing the risk of adverse birth outcomes and leading to adverse physiological health outcomes for children in adulthood [4].American scholar Whitaker R.C. proposed the concept of intergenerational transmission of parent-child health in 1997 [5]. In 1998, Ahlburg found that the correlation coefficient of intergenerational transmission of parent-child obesity was 0.15–0.30 [6]. Later, in 2010, Classen found that the correlation coefficient of intergenerational transmission of mother-child body mass index (BMI) was 0.32–0.38 [7].

Many factors in early life, especially non-genetic factors, might influence intergenerational transmission, but only a few studies have been conducted. On the one hand, genetic factors explain only about 5–10% of intergenerational transmission of BMI, which might indicate a greater influence of non-genetic factors [8]. On the other hand, the influence of genetic factors on intergenerational transmission of overweight and obesity between mothers and infants can be changed to some extent by improving non-genetic factors in early life (Table S1), but there is still a lack of systematic research.

Recently, the construction of risk prediction models for intergenerational transmission of overweight and obesity has been studied globally. In 2013, Dawson J.A. et al. proposed a computational statistics-based model, which considers mating equity and fertility rate, and preliminarily explored intergenerational transmission of overweight and obesity [9]. In 2014, Thomas D.M. et al. proposed a risk prediction model based on genetic effects, which captured the influence of genetic factors on intergenerational transmission of overweight and obesity [10]. In 2016, Huang H. et al. described the prevalence of overweight and obesity based on a mathematical model, which explains infectious and non-infectious factors of overweight and obesity to compare the effectiveness of different interventions [11]. However, none of these models did consider factors related to early life, especially non-genetic factors. At the same time, the risk prediction model for intergenerational transmission of overweight and obesity between mothers and infants has not been systematically investigated based on large birth cohorts.

Considering the above-stated, we studied, from a practical perspective, the intergenerational transmission of overweight and obesity between mothers and infants based on the China Medical University Birth Cohort (CMUBC). We analyzed the status of intergenerational transmission in maternal and infant overweight and obesity and the factors during the pregnancy that might affect the the intergenerational transmission. In term of risk prediction, we focused on two groups. One included women who were already overweight and obese before pregnancy. The other comprised women of all gestational ages, especially those with unclear perceptions of pre-pregnancy weight and those on the verge of being overweight. Second, we focused on the particular period of pregnancy to provide tools for predicting the intergenerational risk of overweight and obesity between mother and their offspring at all stages of pregnancy, including the first, second, and third trimesters. Finally, according to the two application scenarios of pregnancy screening and home self-management, risk prediction models for pregnant women were performed (Fig. S1).

## Methods

The study was conducted based on China Medical University Birth Cohort (CMUBC) [12] and aimed to explore the pathogenesis and prevention of maternal and infant disease longitudinally, especially overweight, obesity, and related metabolic diseases. From April 2018 to November 2021, a total of 1355 pregnancies were enrolled. According to the inclusion and exclusion criteria of this study, 908 pregnant women were included. The inclusion criteria for pregnant women were (1) pregnant women > 18 years old; (2) < 14 weeks of gestation at recruitment; (3) permanent residents of Shenyang who had no plans to move out of Shenyang in the next 3 years; (4) women who understood the project and signed the informed consent. The exclusion criteria included women who has mental illness or infectious diseases, and women who had an abortion during this pregnancy. Inclusion criteria for infants comprised (1) singleton; (2) no birth defects; (3) no meconium aspiration syndrome, congenital metabolic system, and nervous system disease; (4) complete physical measurements throughout early life; (5) delivery at Shengjing Hospital, affiliated with China Medical University (Fig. S2).

### Measures

Pregnant women were instructed to conduct a questionnaire survey by trained research nurses. The questionnaire survey was performed at the first, second, and third trimesters, delivery, 42 days after delivery, 6 months of age, and 12 months of age. The questionnaires during pregnancy mainly included basic information, physical information, personal and family history of illness, history of medications, history of pregnancy and delivery, information on the current pregnancy, information about spouse, physical examination information, clinical examination indicators, auxiliary examination items, factors of lifestyle (smoking, drinking, diet, exercise, sleep, etc.), and psychology (depression and stress).

Dietary patterns during pregnancy were investigated by Food Frequency Questionnaire (FFQ), which had good reliability and validity in China [13, 14]. For each type of food, the respondents were asked how often they consumed it and how much they consumed each time In this study, five dietary patterns were extracted, which were named meat-seafood (high-purine) diet pattern, high-protein diet pattern, body-building dietary pattern, unhealthy diet pattern, and high-carb diet pattern(Table S2).

Physical activity was measured using the Pregnancy Physical Activity Questionnaire (PPAQ) [15]. According to the activity energy consumption (metabolic equivalent, MET), 32 items of the scale were divided into four types of physical activity: resting physical activity, low-intensity physical activity, medium-intensity physical activity, and high-intensity physical activity. Each activity has its corresponding weight coefficient. By multiplying the weight coefficient with the energy consumption value of the activity, the physical activity level can be calculated. At the same time, the physical activity level of pregnant women can be measured from different activity types, such as occupational activity, leisure activity, and housework activity.

Sleep during pregnancy was measured by Pittsburgh Sleep Quality Index (PSQI) [16], mainly to investigate subjective sleep quality in the last month. PSQI consisted of 19 self-evaluation items and five items reported by others. The measurement dimensions mainly included seven aspects: sleep quality, sleep time, sleep duration, sleep efficiency, sleep disorders, hypnotic drug use, and daytime dysfunction. Each dimension is scored from 0 to 3 points, and the sum of the scores of each dimension is the total score of PSQI. The total score ranges from 0 to 21 points. PSQI ≥8 is used as the cut-off value for clinical judgment, and the scale has good reliability and validity, which is suitable for studies.

For the evaluation of psychological status during pregnancy, including pregnancy depression and stress, Edinburgh Postnatal Depression Scale (EPDS) and Pregnancy Pressure Scale (PPS) were used for measurement. EPDS was developed in 1987 by Cox J. et al. and translated by Guo et al., proving to be applicable to pregnant women in China through reliability and validity test [17]. This scale contains ten items, and each item is divided into four grades. The scale suggests a critical value of 10 points as a positive criterion for depression. PPS was compiled by Chen Z. in the 1990s and sinicized and applied by Liying P. et al. [18]. The scale consists of 30 items, and each item is divided into four grades. The total score of the scale ranges from 0 to 90 points. The scale suggests that the standard score of PPS > 0 should be used as the criterion for positive pregnancy stress.

Pre-pregnancy weight refers to the weight within 1 month before pregnancy. Pre-pregnancy BMI was calculated as follows: pre-pregnancy BMI = pre-pregnancy weight (kg)/(height m)^2^, which was classified according to the recommended standard of China Obesity Working Group: low weight (BMI < 18.5 kg/m^2^), normal weight (BMI: 18.5–23.9 kg/m^2^), overweight (BMI: 24–27.9 kg/m^2^), and obesity (BMI ≥28 kg/m^2^) [19].

The gestational weight gain (GWG) is a key risk factor for overweight and obesity during pregnancy. GWG rate (kg/week) as the total GWG divided by the number of gestational weeks at delivery. Based on the IOM, above optimal weight gains are above 16 kg for mothers with normal weight, more than 11.5 kg for prepregnancy overweight mothers and more than 9 kg for prepregnancy obesity mothers, respectively [20].

The weight and length at birth of the infants were recorded from the medical records and then at the age of 12 months postpartum by CMUBC staff, according to a standard protocol. The weight and length were measured with a digital scale and a stadiometer while children were wearing no shoes and light clothes (Seca 416 and 376 +; Seca Corporation, Hamburg, Germany). Z-score method was used to evaluate the physical development level of infants based on the World Health Organization (WHO) child growth reference. Overweight was defined as 1 < BAZ < 2, and obesity was defined as BAZ ≥2 [21].

Blood was collected in the clinical laboratory of Shengjing Hospital of China Medical University during pregnancy. At each stage, 20 ml of fasting venous blood was drawn from the pregnant woman, and plasma and blood cells were separated by centrifugation. The clinical laboratory was responsible for the analysis of blood glucose, triglyceride, cholesterol, high-density lipoprotein cholesterol, low-density lipoprotein cholesterol, apolipoprotein, and other indicators in plasma and issued a diagnostic report.

### Statistical analysis

Data were entered using EpiData 3.1 software [22], descriptive analysis was performed using Stata 15.1 software [23] and prediction models were constructed using R-1.3.959 software [24]. Missing data were filled with multiple interpolation method. The analysis of measurement data was expressed as mean ± standard deviation, and descriptive analysis of counting data was expressed by frequency and percentage. Moreover, the pre-pregnancy overweight and obesity group was matched with the 12-month-old infant overweight and obesity group as the intergenerational transmission group (mothers with overweight/obesity and infants with overweight/obesity, OM-OI), which represented the exposure group. The other three groups (mothers with overweight/obesity and infants without overweight/obesity, OM-NOI, mothers without overweight/obesity and infants without overweight/obesity, NOM-NOI, mothers without overweight/obesity and infants with overweight/obesity, NOM-OI) represented the control group. Furthermore, the results of the two groups (OM-OI and OM-NOI) were compared and statistically analyzed.

In terms of risk prediction, univariate analysis was used for preliminary screening. The preliminary screening of the risk of intergenerational transmission for pregnancy screening and self-management included basic information, physical information, personal and family history of illness, the history of medication, the history of pregnancy and delivery, the information of the current pregnancy, the information of spouse, physical examination information, clinical examination indicators, auxiliary examination items, and factors of lifestyle, psychology, environmental exposure, etc. And then a generalized Logit model was used to screen out pregnancy factors with statistical significance as candidate predictors. According to Akaike’s information criterion (AIC), the model is measured, and model predictors are screened. The principle is that the smaller the AIC value, the better the goodness of fit. Second, multivariable Cox proportional hazard regression models were used to determine the final selected predictors. After integrating the predictors, we draw them in line segments according to the contribution degree of the predictors to the outcome variables. After adding the scores, the total score was obtained, the total score was converted into the probability of the outcome variables, and then the predicted value was obtained. According to the obtained risk probability, this study divided the risk into three levels: high (> 70), medium (30–70), and low (< 30). Finally, the 10-fold cross-validation methods were used for internal verification, and the indexes were discrimination and calibration. Differentiation was represented by an area under the curve (AUC) and receiver operating characteristic (ROC) curve. The degree of calibration was represented by the Hosmer–Lemeshow test and calibration graph.

## Results

A total of 908 pregnant women completed the follow-up survey during pregnancy (Table 1).Among 908 subjects, a total of 833 mother-infant pairs were included by excluding abnormal values of physical indicators after 1-year follow-up during infancy (Fig.S3). The rate of overweight and obesity among infants was 29.1% (Table S3/S4).

**Table 1:** Demographic characteristics of mother-infant pairs in CMUBC

Variable	n	%
Mothers		
Age		
< 25	24	2.6
25–29	305	33.6
30–35	375	41.3
> 35	204	22.5
Nationality		
Han	756	83.3
Others	152	16.7
Educational level		
Junior high school and below	91	10.0
High school and technical secondary school	70	7.7
Bachelor degree/college or above	634	69.8
Postgraduate or above	113	12.5
household income yearly		
< 3	167	18.4
3–5	417	46.0
5–7	86	9.5
> 7	238	26.1
Gravidity		
1	409	45.1
2	274	30.2
≥ 3	225	24.7
Parity		
0	652	71.9
≥ 1	256	28.1
Pre-pregnancy BMI(kg/m^2^)		
< 18.5	61	6.7
18.5 < 23.9	438	48.3
24.0 < 27.9	274	30.2
≥ 28.0	135	14.8
GWG		
Insufficient	112	12.4
Appropriate	344	37.8
Redundancy	452	49.8
Caesarean section		
Yes	426	46.9
No	482	53.1
Gestational age(week)		
< 37	84	9.3
≥ 37	824	90.7
Gestational diabetes		
Yes	212	23.4
No	696	76.3
Gestational hypertension		
Yes	144	15.9
No	774	84.1
Infants		
Sex		
Male	449	49.4
Female	459	50.6
Birth weight		
< 2500	95	10.5
2500–3999	747	82.3
≥ 4000	66	7.2
Growth Level		
GA	50	5.5
AGA	782	86.1
LGA	76	8.4

Maternal overweight and obesity before pregnancy was an independent risk factor for infant overweight and obesity. Compared to their mothers’ normal weight before pregnancy, infants born to mothers with overweight were 180% more likely to be overweight and obese at 12 months of age. Moreover, infants born to obese mothers were 222% more likely to be overweight and obese at 12 months of age.

The status of intergenerational transmission of overweight and obesity between mothers and infants was analyzed. Mothers with overweight/obesity and infants with overweight/obesity (OM-OI), which was called the exposure group. The results showed that there were 166, 270, 76, and 321 cases in the OM-OI, OM-NOI, NOM-NOI, and OM-NOI groups, accounting for 20.0, 32.4, 9.1, and 38.5% of the total population, respectively (Table S5).

Pregnancy variables initially screened were included as independent variables, and then the prediction model with the minimum AIC value was selected (Table 2/S6/S7). The risk prediction model for intergenerational transmission of overweight and obesity between mothers and infants during pregnancy was constructed, which was suitable for pregnancy screening. The model established with the other three groups as the control group: h(t|X) = h_0_(t)exp.(− 0.95 × (Bachelor Degree or above) + 0.75 × (Fasting blood glucose in the second trimester) + 0.89 × (Blood pressure in the third trimester) + 0.80 × (Cholesterol in third trimester) + 0.55 × (Abdominal circumference in third trimester)). The model established with OM-NOI as the control group: h(t|X) = h_0_(t)exp. (− 0.82 × (Bachelor Degree or above) + 0.37 × (Blood glucose 2 hours after taking glucose in the second trimester) + 0.42 × (Blood pressure in the third trimester) + 0.38 × (Cholesterol in third trimester)).

**Table 2:** Multivariate Cox proportional hazard regression models of the risk of intergenerational transmission of overweight and obesity for pregnancy screening^a^

Variable	Other three groups as the control group		OM-NOI as the control group	
	RR(95%CI)*	*P*	RR(95%CI)*	*P*
Educational level				
Junior high school and below	1	–	1	–
High school and technical secondary school	0.60(0.07,1.04)	0.20	0.80(0.30,1.04)	0.12
Bachelor degree/college or above	0.45(0.05,0.80)	0.02	0.74(0.24,0.98)	0.04
FBG in second trimester	1.76(1.22,2.13)	0.008	–	–
Blood glucose 2 h after taking glucose in second trimester	–	–	1.25(1.08,1.34)	0.04
Blood pressure in third trimester	2.89(1.33,3.31)	0.007	1.49(1.08,1.55)	0.005
Cholesterol in third trimester	1.78(1.09,2.22)	0.01	1.26(1.02,1.37)	0.02
Abdominal circumference in third trimester	1.51(1.08,2.55)	0.04	–	–

^a^: adjusted for paternal age, nationality, education level, BMI, and disease history

* (1) RR:Relative Risk; (2) The collinearity diagnosis results showed that there was no severe collinearity

OM:mothers with overweight/obesity before pregnancy;NOM:mothers without overweight/obesity before pregnancy;OI:infants with overweight/obesity; NOI:infants without overweight/obesity

**Fig. 1:**
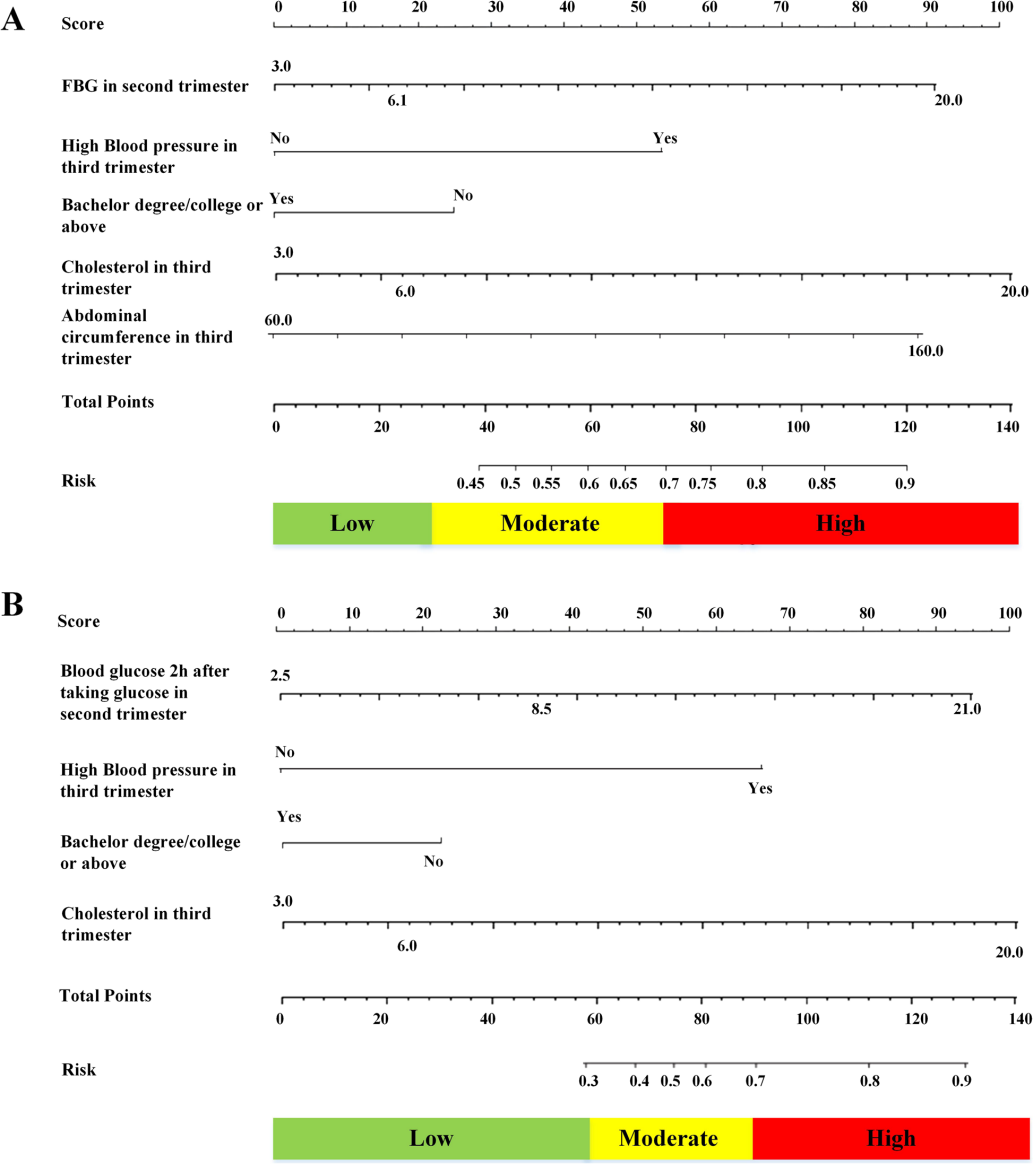
Nomogram of the risk of intergenerational transmission of overweight and obesity for pregnancy screening. **A** Other three groups as the control group. **B** OM-NOI as the control group

**Fig. 2:**
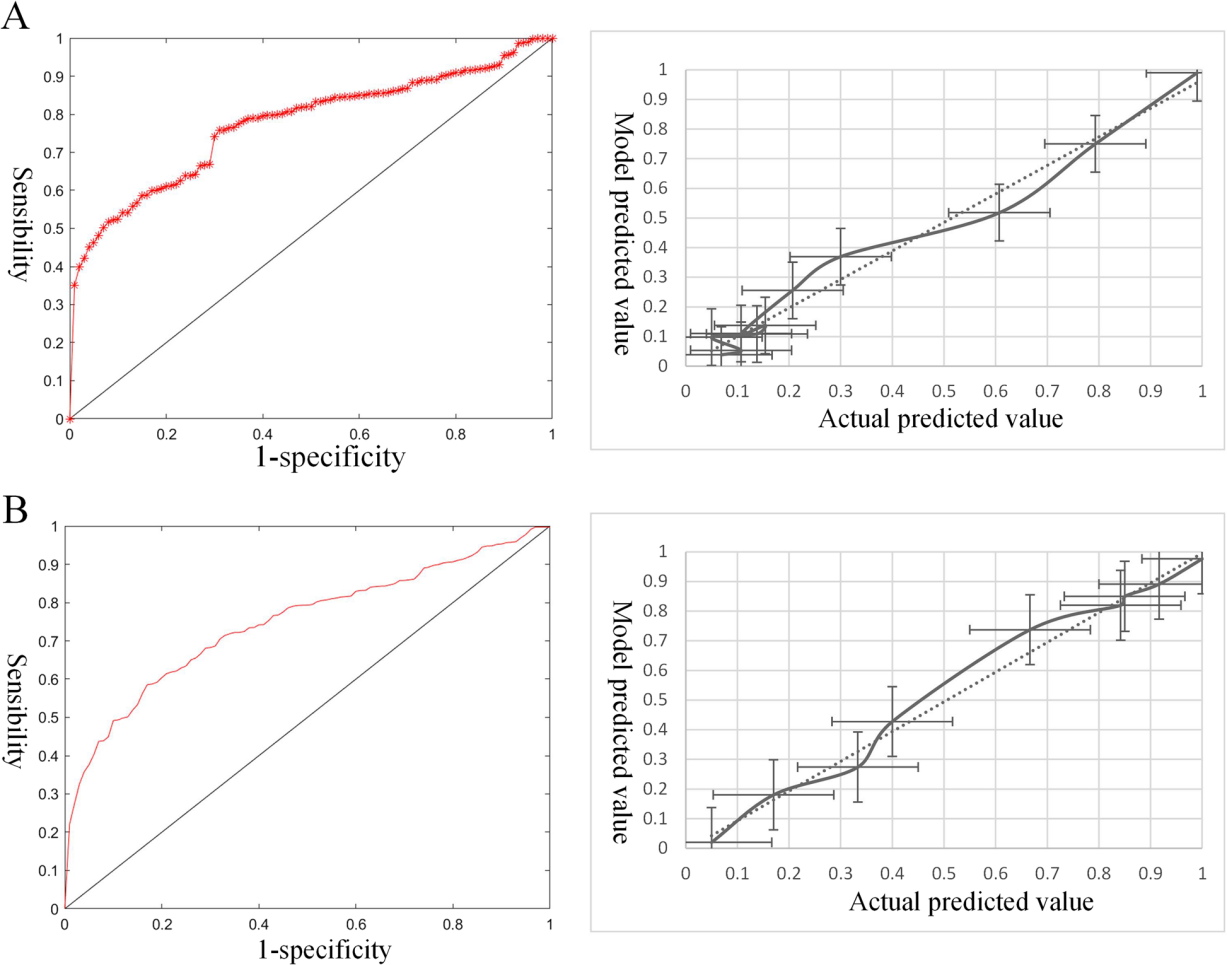
ROC and Calibration curve of the risk of intergenerational transmission of overweight and obesity for pregnancy screening. **A** Other three groups as the control group. **B** OM-NOI as the control group

The nomogram of the risk of intergenerational transmission of overweight and obesity for pregnancy screening is shown in Fig. 1. The AUC of the 10-fold cross-validation methods in the risk of intergenerational transmission of overweight and obesity for pregnancy screening was 0.82 (sensitivity: 0.84; specificity: 0.78) and 0.77 (sensitivity: 0.78; specificity: 0.76), indicating good discrimination. Furthermore, the results showed that the Hosmer-Lemeshow^2^ values were 4.17 (*P* = 0.50 > 0.05) and 6.13 (*P* = 0.42 > 0.05), indicating that the difference between the model-predicted value and the actual predicted value was not statistically significant; thus, the prediction model had good calibration ability (Fig.2).

After taking the other three groups as the control group, four independent variables were included in the final mode (Table 3/S8/S9). The risk prediction model for intergenerational transmission of overweight and obesity between mothers and infants during pregnancy was constructed, which was suitable for self-management. The model established with the other three groups as the control group: h(t|X) = h_0_(t)exp. (0.98 × (Sedentary >18METs) + 0.88 × (Sleep index≥8) + 0.81 × (Unhealthy eating patterns Q3/Q4) + 0.90 × (Unhealthy eating patterns Q4/Q4) + 0.85 × (Depression)). The model established with OM-NOI as the control group: h(t|X) = h_0_(t)exp. (− 2.11 × (Housework 44.3-54METs)-1.47 × (Housework >54METs) + 0.67 × (Sleep index≥8) + 0.65 × (Unhealthy eating patterns Q3/Q4) + 0.83 × (Unhealthy eating patterns Q4/Q4) + 0.71 × (Depression)).

**Table 3:** Multivariate Cox proportional hazard regression models of the risk of intergenerational transmission of overweight and obesity for self- management applications ^a^

Variable	Other three groups as the control group		OM-NOI as the control group	
	RR(95%CI)*	*P*	RR(95%CI)*	*P*
**First trimester**				
Sedentary				
< 10.5	1	–	–	–
10.6-	1.45(0.52,3.07)	0.42	–	–
13.5-	1.58(0.63,2.51)	0.40	–	–
18-	1.95(1.52,2.50)	0.03	–	–
Housework				
< 39.75	–	–	1	–
39.8-	–	–	0.46(0.12,1.06)	0.31
44.3-	–	–	0.22(0.05,0.73)	0.03
54-	–	–	0.39(0.10,0.98)	0.04
**Second trimester**				
Sleep Quality Index (≥8)	2.21(1.26,3.08)	0.02	1.43(1.07,1.53)	< 0.001
Unhealthy dietary patterns				
Q1	1	–	1	–
Q2	1.44(0.53,2.70)	0.51	1.25(0.76,1.51)	0.32
Q3	2.25(1.12,2.97)	0.003	1.35(1.12,1.52)	0.007
Q4	2.90(2.07,3.30)	0.003	1.51(1.38,1.57)	0.004
**Third trimester**				
Depression	2.40(1.42,3.23)	0.01	1.42(1.12,1.55)	0.02

^a^: adjusted for paternal age, nationality, education level, BMI, and disease history

* (1) RR:Relative Risk; (2)The collinearity diagnosis results showed that there was no severe collinearity

OM:mothers with overweight/obesity before pregnancy;NOM:mothers without overweight/obesity before pregnancy;OI:infants with overweight/obesity; NOI:infants without overweight/obesity

**Fig. 3:**
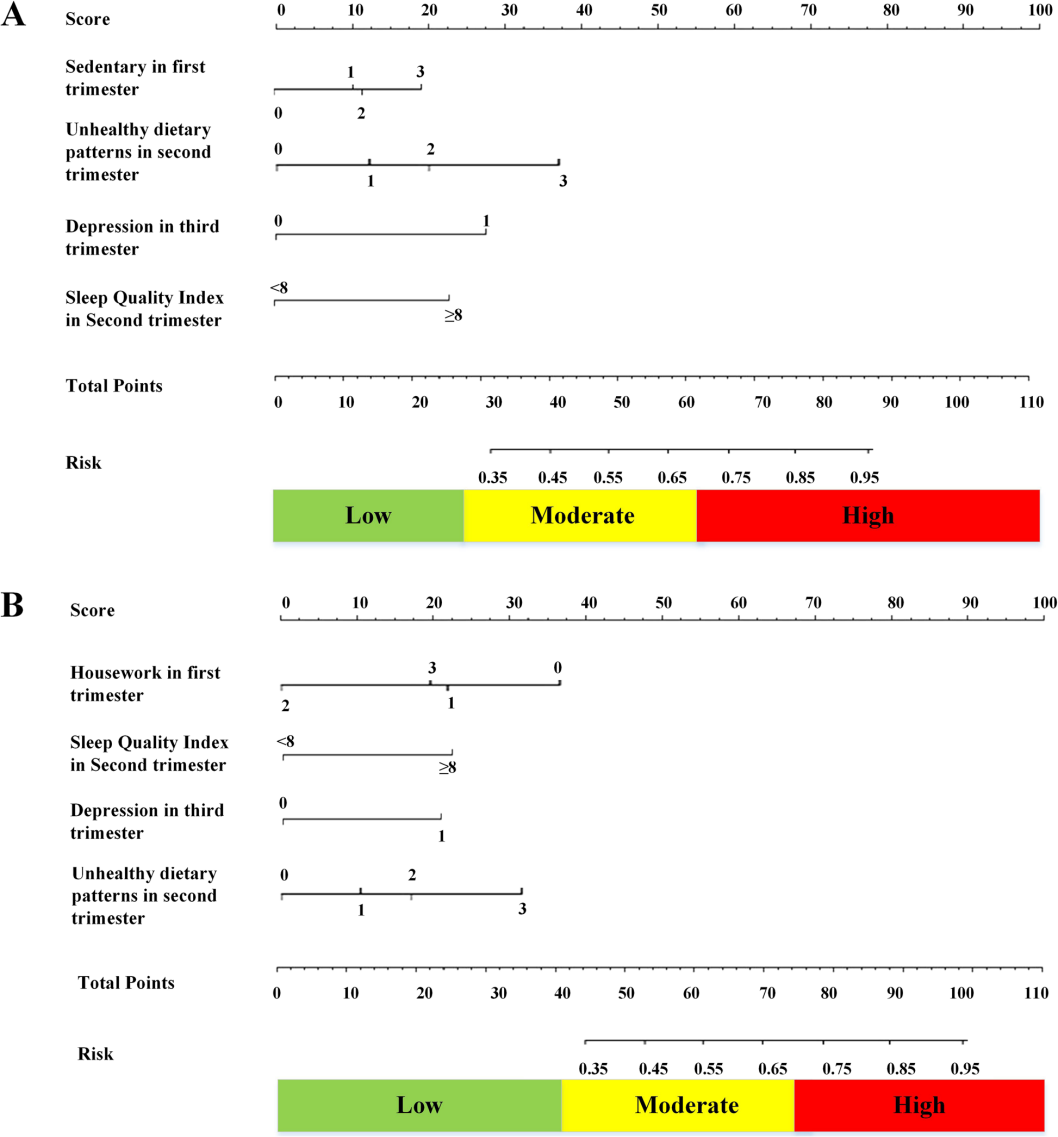
Nomogram of the risk of intergenerational transmission of overweight and obesity for self- management. **A** Other three groups as the control group. **B** OM-NOI as the control group

**Fig. 4:**
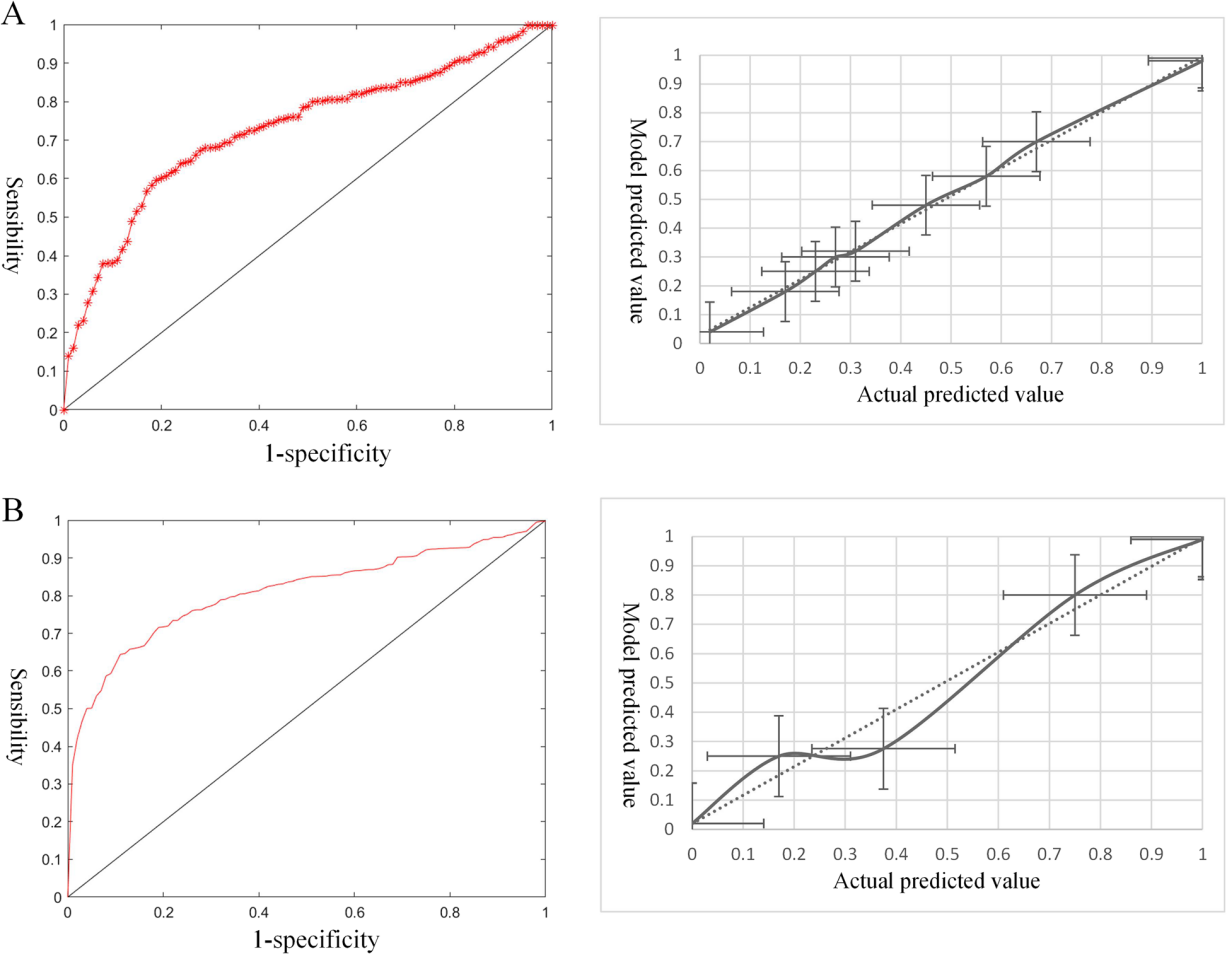
ROC and Calibration curve of the risk of intergenerational transmission of overweight and obesity for self- management. **A** Other three groups as the control group. **B** OM-NOI as the control group

The nomogram of the risk of intergenerational transmission of overweight and obesity for self-management is shown in Fig. 3. The AUC of the 10-fold cross-validation methods in the risk of intergenerational transmission of overweight and obesity for self-management was 0.75 (sensitivity: 0.72; specificity: 0.76) and 0.76 (sensitivity: 0.77; specificity: 0.80), indicating good discrimination. Furthermore, the results showed that the Hosmer-Lemeshow^2^ values were 3.81 (*P* = 0.27 > 0.05) and 4.33 (*P* = 0.30 > 0.05); therefore, the risk prediction model for self-management also had better calibration ability (Fig.4).

## Discussion

### Main findings

Based on a population-based birth cohort database, we conducted the study on intergenerational transmission of overweight and obesity between mothers and infants during pregnancy. The risk predictions were performed for two populations, one period, and two application scenarios (pregnancy screening and home self-management). Risk predictions for pregnancy screening should focus on educational level, blood glucose in the second trimester, blood pressure/cholesterol/abdominal circumference in the third trimester, etc. Risk predictions for self-management should focus on physical activity in the first trimester, dietary patterns in the second trimester, and psychological status in the third trimester.

Identifying the influencing factors in early life is important to prevent the occurrence and development of intergenerational transmission.

### Comparison with previous studies

The origin of childhood overweight and obesity can be traced back to early life, thus, the first 1000 days of life. Recent studies have found that maternal pre-pregnancy BMI is a powerful predictor of overweight and obesity in offspring, which is manifested in the intergenerational transmission of overweight and obesity between mothers and infants. Identifying the influencing factors in early life is important to prevent the occurrence and development of intergenerational transmission. In this study, the pregnancy predictors of intergenerational transmission of maternal and infant overweight and obesity for pregnancy screening were explored.

This study found that socioeconomic status, especially educational level, is an important factor influencing intergenerational transmission of overweight and obesity between mothers and infants, which is consistent with the results of previous cohort studies [25]. Although the mechanisms are not clear, lower literacy might play a role through biological risk (developmental programs and fetal growth restriction) and social risk chains (transformation of health knowledge into a healthy lifestyle in a social model) [26, 27];for example, women with lower education levels, especially during pregnancy, are more likely to engage in unhealthy lifestyle behaviors.

Blood glucose is one of the recognized risk factors for intergenerational transmission. Our study found that the risk of intergenerational transmission of overweight and obesity between mothers and infants was associated with an increase in blood glucose in the second trimester, which is consistent with previous research results [28, 29]. At the same time, previous studies highlight the correlation between maternal hyperglycemia and particular time points in pregnancy and demonstrate that even in the absence of diabetes, blood glucose is a high-risk factor .

Studies showed that the relationships between maternal lipids and the early life growth of offspring are different due to the pre-pregnancy weight status, meaning that the lipid status during pregnancy is an influential factor in the intergenerational transmission of mothers and infants with obesity. The Healthy Start Study in the United States found a positive association between cholesterol in the third trimester and BMI/fat mass in early life only in women who were overweight/obese before pregnancy [30]. These findings point to the need to further explore how maternal BMI, independently or in interaction with metabolic markers, leads to fuel-mediated overnutrition, which, in turn, affects the growth and development of offspring.

Only a few studies have been conducted on the relationship between blood pressure during pregnancy and intergenerational transmission of overweight and obesity, but some studies still show that there is a positive correlation between the two [31, 32]. High blood pressure during pregnancy is a well-known cause of intrauterine growth restriction, especially in the third trimester [33]. Increased vascular resistance of the umbilical artery in the third trimester reflects placental dysfunction. While this is associated with slow intrauterine growth and small size at birth, it can lead to higher BMI in early life and childhood [34, 35].

Abdominal circumference during pregnancy can effectively assess fetal growth and development. We found that abdominal circumference in the third pregnancy is a risk factor for intergenerational transmission of mothers and infants with overweight and obesity. S1000 study in Africa found that abdominal circumference is a key intermediary factor for intergenerational transmission [36]. Excessive abdominal circumference during pregnancy is an important predictor of adverse birth outcomes, highlighting the specific impact of fat accumulation on obesity, which increases the risk of long-term obesity and non-communicable diseases. It has potential value in the identification of risks during pregnancy..

The influence of genetic factors on intergenerational transmission of overweight and obesity between mothers and infants can be changed to some extent by improving non-genetic factors in early life [37]. Therefore, we found that diet (unhealthy eating patterns), physical activity (sedentary lifestyle and housework), sleep quality, and psychology (depression) were predictors of intergenerational transmission of overweight and obesity between mothers and infants for self-management.

Diet during pregnancy, as a recognized factor affecting the intergenerational transmission of overweight and obesity, has been investigated to a relatively limited extent. Diet during pregnancy can be planned for fetal development through its effect on the intrauterine environment. When the intrauterine environment is exposed to metabolic changes causing metabolic stress, fetal development and metabolic adaptation may be altered through epigenetic changes or interference with homeostasis control mechanisms, leading to an increased risk of overweight and obesity in the offspring. The results of our study found that the risk of intergenerational transmission of overweight and obesity was nearly six times higher in women with unhealthy dietary patterns during the second trimester. A study by Martin C.L. et al. on dietary patterns during pregnancy and intergenerational transmission of overweight and obesity between mothers and infants found that unhealthy dietary patterns were significant risk factors (RR: 1.83; 95% CI: 1.02, 3.28) intergenerational transmission of overweight and obesity in infants [38]. In contrast, a prospective study of Dutch mothers and their offspring, after adjusting for other confounding factors, demonstrated no significant association between diet patterns during pregnancy and intergenerational transmission of overweight and obesity between mothers and infants [39].

Physical activity during pregnancy can impact the growth of their offspring. Our study found that physical activity in early pregnancy, such as a sedentary lifestyle and housework, is one of the key factors influencing intergenerational transmission of overweight and obesity. These results are consistent with the Canadian study showing that regular physical activity before the second trimester leads to lower body weight and fat mass in the offspring. The reason might be that regular exercise during pregnancy can reduce inflammation and oxidative stress, which, in turn, can lead to a temporary decrease in the maternal blood flow and the availability of nutrients in the uterus and placenta, thereby reducing the delivery of excess nutrients to the fetus. However, current knowledge of the relationship between the pattern/intensity of physical activity and intergenerational transmission of overweight and obesity between mothers and infants is still inconsistent [40, 41].

Regarding sleep during pregnancy, especially in the second and third trimesters, mothers are more likely to have sleep problems, and these problems are more prominent in mothers who are overweight and obese before pregnancy. Our findings suggest that poor sleep quality leads to an increased risk of intergenerational transmission of overweight and obesity. Avivit Brener and other scholars showed that with poor sleep quality during pregnancy, the risk of giving birth to low-birth-weight infants was greater. These infants were more likely to develop catch-up growth in early life and be overweight and obese by the age of 3 years. The above-mentioned phenomenon is more likely to occur in mothers who are overweight and obese before pregnancy [42]. Meng M. et al. found that newborns born to overweight and obese mothers with insufficient sleep time in the second and third trimesters had lower leptin levels and higher triglyceride levels in the cord blood, thus, affecting their development [43].

Although depression during pregnancy is associated with an increased risk of pregnancy complications and adverse birth outcomes, there has been little research on infant growth, especially focusing on the offspring of overweight and obese women before pregnancy. Karen A. et al. showed that overweight and obese women with prenatal depression had higher levels of corticotropin-releasing hormone than non-depressed women, which would increase the risk of short height and central obesity in their offspring [44]. However, an authoritative meta-analysis showed that depression during pregnancy did not lead to overweight and obesity in the offspring, but the interaction between maternal lifestyle factors (diet, exercise, sleep, etc.) and pre-pregnancy BMI affected the intrauterine environment and then caused long-term effects on the metabolic environment of the offspring [45]. Thus, depression during pregnancy mostly affects overweight and obesity in offspring by influencing birth outcomes and lifestyle factors, and more attention should be paid to mechanisms and mediating factors in intergenerational transmission to avoid missing key directions.

### Evaluation of risk prediction models

In view of the fact that few studies have systematically explored the pregnancy risk prediction model of intergenerational transmission, we developed such a model based on the above-mentioned predictors. Our study conducted risk prediction for two application populations. First, we focused on two groups. One was women who were already overweight and obese before pregnancy. By using our risk prediction models to understand their own risk index, we hope to allow clinicians to minimize exposure of offspring to obesogenic environments and then break the vicious cycle of intergenerational transmission of overweight and obesity. The other group comprised women of all gestational ages, especially those with unclear perceptions of pre-pregnancy weight and those on the verge of being overweight. This model humanely considered women at different weight levels. It can promote the realization of the importance of weight control among all pregnant women to detect and control risk factors at an early stage. We also used the model to predict the risk of intergenerational transmission based on two application scenarios. Medical staff in charge of pregnancy examination can use it to assess mothers more accurately from a professional perspective. For pregnant women, self-management can be more targeted and evidence-based using various aspects such as lifestyle, environmental factors, and behavioral factors. The two risk prediction models involved in this study had high efficacy, providing practical and effective assessment tools for preventing and controlling intergenerational transmission of overweight and obesity during pregnancy.

### Strengths and limitations

One strength of this study is that it relied on a large sample database of the birth cohort. The second strength is that it focused on maternal and infant obesity and introduced new evidence and perspective regarding intergenerational transmission. Although our study presents novelty in terms of preventing childhood obesity, there were some limitations. First, the questionnaires were answered by mothers; hence, we could not completely eliminate bias. Second, the proportion of missing values was relatively high, but we used the method of multiple imputation techniques to reduce their influence. Finally, we used 10-fold cross-validation to internally verify the prediction model. However, we recommend independent external validation to evaluate the effect of this model when generalized to other birth cohorts.

### Supplementary Information

**Additional file 1.**

**Additional file 2.**

**Additional file 3.**

**Additional file 4.**

## Data Availability

The data of this study is available from the corresponding authors on reasonable request.
