# Supplementary material for: The risk prediction of intergenerational transmission of overweight and obesity between mothers and infants during pregnancy
Source: BMC Pregnancy Childbirth. 2024 Jan 23;24:74. doi: 10.1186/s12884-024-06268-7 (PMC10804797; doi:10.1186/s12884-024-06268-7)
Supplement: Supplementary file 1 — Additional file 1. [file 12884_2024_6268_MOESM1_ESM.docx]

Table S1: Factors influencing the intergenerational transmission of overweight and obesity between mothers and infants.

| Author and year | Country | Study design | Sample | Factors |
| --- | --- | --- | --- | --- |
| Anna S. et al,^[1]^ 2017 | New Zealand | Birth cohort | 1043 | Mental factors |
| Camilla S.M. et al,^[2]^ 2017 | Denmark | Birth cohort | 29374 | Socioeconomic status, maternal smoking, pregnancy weight gain |
| Chun-Ying L. et al,^[3]^ 2016 | China | Birth cohort | 2727 | Demographic factors, dietary patterns, physical activity, anthropometric indicators and clinical parameters |
| Costa-Font J. et al,^[4]^ 2013 | UK | Birth cohort | 13358 | Socioeconomic status (education level, income inequality) |
| Crume T.L. et al,^[5]^ 2011 | USA | Birth cohort | 461 | Gestational diabetes |
| Cuilin Z. et a,^[6]^ 2017 | USA | Birth cohort | 2802 | Fetal growth and development (ultrasound, fetal humerus length, femur length, double parietal diameter, head circumference, and abdominal circumference) |
| Klodian D. et al,^[7]^ 2018 | USA | Birth cohort | 5701 | Lifestyle (smoking, diet, physical activity) |
| L.H. Lumey. et al,^[8]^ 2007 | Germany | Birth cohort | 3307 | Pregnancy nutrition |
| Mary K.et al,^[9]^ 2016 | Ireland | Birth cohort | 281 | Maternal lifestyle, anthropometric indicators and clinical parameters |
| Pilar N. et al,^[10]^ 2019 | USA | Birth cohort | 1082 | Dietary pattern |
| Ranjit M.A. et al,^[11]^ 2009 | India | Cross-sectional study | 321 | Disease history, clinical indicators |
| Stephanie V. et al, ^[12]^ 2020 | UK | Birth cohort | 304 | SITAR fetal growth index (femur length, abdominal circumference, bi-parietal diameter, and head circumference) |
| Uttara P. et al,^[13]^ 2017 | Malaysia | Birth cohort | 9207 | Metabolic factors (blood pressure, blood sugar, etc.) |
| S. Swaminathan. et al,^[14]^ 2013 | India | Cross-sectional study | 325 | Diet, physical activity, sleep |

References (Table S1)

1. Serlachius A, Pulkki-R, Back L, et al. Does high optimism protect against the inter-generational transmission of high BMI? The Cardiovascular Risk in Young Finns Study. *J Psychosom Res*. 2017;100:61.
2. Morgen CS, Ngquist L, Baker JL, et al. Prenatal risk factors influencing childhood BMI and overweight independent of birth weight and infancy BMI: a path analysis within the Danish National Birth Cohort. *Int J Obes*. 2017;42(4).
3. Chun-Ying L, Lin WT, Sharon T, et al. Association of parental overweight and cardiometabolic diseases and pediatric adiposity and lifestyle factors with cardiovascular risk factor clustering in adolescents. *Nutrients*. 2016;8(9).
4. Costa-Font J, Gil J. Intergenerational and socioeconomic gradients of child obesity. *Soc Sci Med*. 2013;93(5):29-37.
5. Crume TL, Ogden L, West NA, et al. Association of exposure to diabetes in utero with adiposity and fat distribution in a multiethnic population of youth: the Exploring Perinatal Outcomes among Children (EPOCH) Study. *Diabetologia*. 2011;54(1):87-92.
6. Zhang C, Hediger ML, Albert PS, et al. Association of maternal obesity with longitudinal ultrasonographic measures of fetal growth: Findings from the NICHD fetal growth studies–singletons. *Jama Pediatr*. 2017.
7. Dhana K, Zong G, Yuan C, et al. Lifestyle of women before pregnancy and the risk of offspring obesity during childhood through early adulthood. *Int J Obes*. 2018;14(1):67-75.
8. Lumey L, Stein AD, Kahn HS, et al. Cohort profile: The Dutch Hunger Winter Families study. *Int J Epidemiol*. 2007;36(6):1196-1204.
9. Horan MK, Donnelly JM, Mcgowan CA. The association between maternal nutrition and lifestyle during pregnancy and 2-year-old offspring adiposity: analysis from the ROLO study. *J Public Health*. 2016;24(5):427-436.
10. Navarro P, Shivappa N, Hebert JR, et al. Intergenerational associations of dietary inflammatory index with birth outcomes and weight status at age 5 and 9: Results from the lifeways cross generation cohort study. *Pediatr Obesity*. 2019;15(3).
11. Anjana RM, Lakshminarayanan S, Deepa M, et al. Parental history of type 2 diabetes mellitus, metabolic syndrome, and cardiometabolic risk factors in Asian Indian adolescents. *Metab Clin Exp*. 2009;58(3):344-350.
12. Wrottesley SV, Prioreschi A, Norris SA. The association between fetal growth and neonatal adiposity in urban South African infants. *Pediatr Obesity*. 2020;35(10):1425-1433.
13. Partap U, Young EH, Allotey P, et al. Anthropometric and cardiometabolic risk factors in parents and child obesity in Segamat, Malaysia. *Int J Epidemiol*. 2017;(5):1523.
14. Swaminathan S, Thomas T, Yusuf S, et al. Clustering of diet, physical activity and overweight in parents and offspring in South India. *Eur J Clin Nutr*. 2013;67(2):128-134.

Table S2: Factor analysis of FFQ during pregnancy.

| **Dietary pattern** | **Main food** | **Factor loading** | **Accumulative contribution rate (%)** |
| --- | --- | --- | --- |
| Meat-seafood (high purine) dietary pattern | Red meat | 0.528 | 11.17 |
|  | Chicken | 0.612 |  |
|  | Haslet | 0.596 |  |
|  | River fish | 0.637 |  |
|  | marine fish | 0.792 |  |
|  | Shrimp and crab | 0.655 |  |
|  | Seaweeds | 0.605 |  |
| High-protein dietary pattern | Dairy product | 0.764 | 21.11 |
|  | Milk | 0.733 |  |
|  | Eggs | 0.507 |  |
|  | Bean products | 0.455 |  |
| Body-building dietary pattern | Vegetable | 0.827 | 39.47 |
|  | Fruit | 0.844 |  |
|  | Nut | 0.454 |  |
| Unhealthy dietary pattern | Dessert | 0.681 | 48.08 |
|  | Fried food | 0.743 |  |
|  | Sugared beverages | 0.773 |  |
| High-carb dietary pattern | Rice | 0.692 | 56.53 |
|  | Cooked wheaten food | 0.688 |  |
|  | Coarse cereals | 0.735 |  |
|  | Potato | 0.612 |  |

Table S3: Comparative analysis of offspring with overweight and obesity at 12 months when mothers were overweight and obesity before pregnancy

| Variable | Normal  (n=591) | | | Overweight  (n=201) | |  | Obesity  (n=41) | |  | Overweight&Obesity  (n=242) | | χ^2a^ | *P*^a^ | χ^2b^ | *P^b^* | χ^2c^ | *P*^c^ |
| --- | --- | --- | --- | --- | --- | --- | --- | --- | --- | --- | --- | --- | --- | --- | --- | --- | --- |
|  | n | % |  | n | % |  | n | % |  | n | % |  |  |  |  |  |  |
| Pre-pregnancy BMI(kg/m2) |  |  |  |  |  |  |  |  |  |  |  | 13.95 | 0.003 | 42.65 | <0.001 | 43.65 | <0.001 |
| <18.5 | 43 | 7.2 |  | 6 | 3.0 |  | 5 | 3.6 |  | 7 | 2.9 |  |  |  |  |  |  |
| 18.5-23.9 | 278 | 47.0 |  | 68 | 33.6 |  | 5 | 3.6 |  | 69 | 28.5 |  |  |  |  |  |  |
| 24-27.9 | 187 | 31.6 |  | 86 | 43.0 |  | 23 | 76.0 |  | 116 | 47.9 |  |  |  |  |  |  |
| ≥28 | 83 | 14.2 |  | 41 | 20.4 |  | 8 | 19.8 |  | 50 | 20.7 |  |  |  |  |  |  |

a:Overweight vs. Normal ; b:Obesity vs. Normal; c:Overweight&Obesity vs. Normal.

Table S4: Linear relationship between overweight and obesity of mothers before pregnancy and overweight and obesity of their offspring at 12 months ^a^

| Variable | Overweight | | Obesity | | Overweight&Obesity | |
| --- | --- | --- | --- | --- | --- | --- |
|  | OR | 95%CI | OR | 95%CI | OR | 95%CI |
| Pre-pregnancy BMI(kg/m2) |  |  |  |  |  |  |
| <18.5 | 0.97 | 0.76,1.18 | 0.73 | 0.65,0.81 | 0.89 | 0.79,1.00* |
| 18.5-23.9 | 1 | Ref | 1 | Ref | 1 | Ref |
| 24-27.9 | 1.66 | 1.02,2.70* | 4.57 | 3.27,9.87** | 2.80 | 1.61,4.88** |
| ≥28 | 1.87 | 1.02,3.43* | 6.50 | 6.04,13.18** | 3.22 | 2.07,5.01** |

a:adjusting for maternal age, nationality, education level, household income yearly,Gravidity ,Parity, delivery mode, gestational age, gestational disease and infant’ sex.

* *P*<0.05; ** *P*<0.001

Table S5: Intergenerational transmission of overweight and obesity [n(%)]

| Variable | OM | NOM |
| --- | --- | --- |
| OI | 166(20.0) | 76(9.1) |
| NOI | 270(32.4) | 321(38.5) |

OM:mothers with overweight/obesity before pregnancy ;NOM:mothers without overweight/obesity before pregnancy ;OI:infants with overweight/obesity; NOI:infants without overweight/obesity

Table S6: Univariate analysis of the risk of intergenerational transmission of overweight and obesity for pregnancy screening^a^

| **variable** | **Other three groups as the control group** | | | **OM-NOI as the control group** | | |
| --- | --- | --- | --- | --- | --- | --- |
|  | **OR** | **95%CI** | ***P*** | **OR** | **95%CI** | ***P*** |
| **Preparation for pregnancy** |  |  |  |  |  |  |
| Age | 1.05 | 1.01,1.09 | 0.01 | 1.05 | 1.00,1.10 | 0.05 |
| Educational level |  |  |  |  |  |  |
| Junior high school and below | 1 | Ref | - | 1 | Ref | - |
| High school and technical secondary school | 0.50 | 0.02,0.98 | 0.04 | 0.40 | 0.08,0.72 | 0.03 |
| Bachelor degree/college or above | 0.20 | 0.11,0.35 | <0.001 | 0.16 | 0.12,0.20 | 0.01 |
| History of preterm birth | 4.08 | 1.27,13.03 | 0.02 | 2.03 | 0.91,3.15 | 0.07 |
| History of pregnancy complications | 2.50 | 1.00,6.34 | 0.06 | - | - | - |
| **First trimester** |  |  |  |  |  |  |
| Assisted reproductive technology | 1.55 | 1.00,2.58 | 0.05 | 2.87 | 1.15,7.19 | 0.02 |
| **Second trimester** |  |  |  |  |  |  |
| Gestational diabetes | 1.80 | 1.06,3.08 | 0.04 | - | - | - |
| Gestational hypertension | 1.95 | 1.38,2.77 | <0.001 | 2.92 | 1.56,4.30 | <0.001 |
| Abdominal circumference | 1.06 | 1.04,1.08 | <0.001 | 1.03 | 1.00,1.05 | 0.05 |
| FBG(fasting blood-glucose) | 1.65 | 1.28,2.13 | <0.001 | 1.37 | 1.03,1.71 | 0.05 |
| Blood glucose 1h after taking glucose | 1.13 | 1.03,1.25 | 0.01 | - | - | - |
| Blood glucose 2h after taking glucose | 1.12 | 1.02,1.24 | 0.06 | 1.15 | 1.00,1.31 | 0.05 |
| **Third trimester** |  |  |  |  |  |  |
| Blood pressure | 3.45 | 1.41,5.49 | 0.01 | 1.78 | 1.09,2.48 | 0.04 |
| Abdominal circumference | 1.03 | 1.00,1.06 | 0.01 | 1.03 | 1.00,1.06 | 0.05 |
| Cholesterol | 1.38 | 1.93,2.04 | 0.01 | 1.59 | 1.03,2.17 | 0.04 |
| Triglyceride | 1.82 | 1.52,2.00 | 0.003 | 1.77 | 1.15,2.38 | 0.04 |
| Apolipoprotein A | 2.80 | 1.22,6.40 | 0.02 | 2.67 | 0.80,8.87 | 0.11 |
| Fetal humerus length | 0.66 | 0.36,1.23 | 0.19 | 0.53 | 0.24,1.19 | 0.12 |
| Calcium | 2.36 | 1.20,4.63 | 0.01 | - | - | - |
| Iron | 2.75 | 1.16,6.51 | 0.02 | - | - | - |
| Antibiotics | 16.40 | 1.73,35.84 | 0.02 | - | - | - |
| Hormone drugs | 7.46 | 2.62,21.22 | <0.001 | 6.00 | 1.80,34.24 | 0.01 |

a:adjusted for paternal age, nationality, education level, BMI, and disease history.

OM:mothers with overweight/obesity before pregnancy ;NOM:mothers without overweight/obesity before pregnancy ;OI:infants with overweight/obesity; NOI:infants without overweight/obesity.

Table S7: Generalized Logit analysis of the risk of intergenerational transmission of overweight and obesity for pregnancy screening

| **Variable** | **Other three groups as the control group** | | **OM-NOC as the control group** | |
| --- | --- | --- | --- | --- |
|  | **OR(95%CI)** | **P** | **OR(95%CI)** | **P** |
| **Preparation for pregnancy^a^** |  |  |  |  |
| Educational level |  |  |  |  |
| Junior high school and below | 1(Ref) | - | 1(Ref) | - |
| High school and technical secondary school | 0.30(0.09,0.55) | 0.01 | 0.22(0.01,0.41) | 0.01 |
| Bachelor degree/college or above | 0.16(0.06,0.32) | <0.001 | 0.12(0.02,0.39) | 0.005 |
| Age | 1.05(1.00,1.11) | 0.04 | - | - |
| History of preterm birth | 5.75(1.45,22.73) | 0.01 | - | - |
| **Second** **trimester ^c^** |  |  |  |  |
| Abdominal circumference | 1.05(1.03,1.08) | <0.001 | - | - |
| FBG(fasting blood-glucose) | 1.41(1.06,1.87) | 0.02 | - | - |
| Blood glucose 2h after taking glucose | - | - | 1.20(1.03,1.42) | 0.03 |
| **Third trimester ^d^** |  |  |  |  |
| Blood pressure | 3.14(1.34,4.97) | <0.001 | 2.17(1.58,2.86) | <0.001 |
| Cholesterol | 2.02(1.22,3.35) | 0.01 | 1.82(1.06,3.11) | 0.03 |
| Abdominal circumference | 1.09(1.00,1.19) | 0.05 | 1.21(1.03,1.42) | 0.02 |
| Apolipoprotein A | 1.52(1.12,2.02) | 0.01 | - | - |

a adjusted for paternal age, nationality, education level, BMI, and disease history.

B adjusted for statistically significant factors in paternal information and preparation for pregnancy；

c adjusted for statistically significant factors in paternal information , preparation for pregnancy and first trimester；

d adjusted for statistically significant factors in paternal information , preparation for pregnancy, first trimester and second trimester；

OM:mothers with overweight/obesity before pregnancy ;NOM:mothers without overweight/obesity before pregnancy ;OI:infants with overweight/obesity; NOI:infants without overweight/obesity.

Table S8: Univariate analysis of the risk of intergenerational transmission of overweight and obesity for home management applications^a^

| Variable | Other three groups as the control group | | |  | OM-NOC as the control group | | |
| --- | --- | --- | --- | --- | --- | --- | --- |
|  | **OR** | **95%CI** | ***P*** |  | **OR** | **95%CI** | ***P*** |
| **First trimester** |  |  |  |  |  |  |  |
| Sedentary |  |  |  |  |  |  |  |
| <10.5 | 1 | Ref | - |  | - | - | - |
| 10.6- | 1.33 | 0.48,1.44 | 0.51 |  | - | - | - |
| 13.5- | 1.10 | 0.95,1.25 | 0.24 |  | - | - | - |
| 18- | 3.34 | 2.11,5.29 | <0.001 |  | - | - | - |
| Housework |  |  |  |  |  |  |  |
| <39.75 | - | - | - |  | 1 | Ref | - |
| 39.8- | - | - | - |  | 0.84 | 0.43,1.63 | 0.61 |
| 44.3- | - | - | - |  | 0.48 | 0.26,0.89 | 0.02 |
| 54- | - | - | - |  | 0.40 | 0.35,0.45 | 0.01 |
| **Second trimester** |  |  |  |  |  |  |  |
| Unhealthy dietary patterns |  |  |  |  |  |  |  |
| Q1 | 1 | Ref | - |  | 1 | Ref | - |
| Q2 | 2.49 | 1.38,4.54 | 0.003 |  | 1.52 | 1.12,1.92 | 0.002 |
| Q3 | 4.08 | 2.21,5.95 | <0.001 |  | 4.63 | 2.75,6.52 | <0.001 |
| Q4 | 6.55 | 3.24,9.96 | <0.001 |  | 5.25 | 3.14,7.46 | <0.001 |
| Low-intensity(1.6-2.9 METs) PA |  |  |  |  |  |  |  |
| <31.5 | 1 | Ref | - |  | 1 | Ref | - |
| 31.6- | 0.76 | 0.48,1.22 | 0.26 |  | 0.88 | 0.70,1.06 | 0.07 |
| 40.5- | 0.38 | 0.23,0.63 | <0.001 |  | 0.73 | 0.51,1.28 | 0.21 |
| 47.25- | 0.45 | 0.28,0.72 | 0.001 |  | 0.53 | 0.28,0.99 | 0.04 |
| Occupation |  |  |  |  |  |  |  |
| <24 | 1 | Ref | - |  | 1 | Ref | - |
| 24- | 0.98 | 0.83,1.17 | 0.10 |  | 0.95 | 0.76,1.20 | 0.09 |
| 28.5- | 0.72 | 0.47,0.93 | 0.03 |  | 0.69 | 0.40,0.95 | 0.03 |
| 32.3- | 0.54 | 0.33,0.70 | <0.001 |  | 0.50 | 0.30,0.65 | <0.001 |
| Housework |  |  |  |  |  |  |  |
| <39.75 | - | - | - |  | 1 | Ref | - |
| 39.8- | - | - | - |  | 0.79 | 0.43,1.47 | 0.46 |
| 44.3- | - | - | - |  | 0.50 | 0.26,0.96 | 0.04 |
| 54- | - | - | - |  | 1.15 | 0.57,2.33 | 0.69 |
| Sleep Quality Index (≥8) | 2.85 | 1.99,4.10 | <0.001 |  | 2.13 | 1.28,3.55 | 0.004 |
| **Third trimester** |  |  |  |  |  |  |  |
| Depression | 1.93 | 1.37,2.72 | <0.001 |  | 1.57 | 1.02,2.13 | 0.05 |

a:adjusted for paternal age, nationality, education level, BMI, and disease history.

* PA:physical activity

Table S9: Generalized Logit analysis of the risk of intergenerational transmission of overweight and obesity for home management applications

| Variable | Other three groups as the control group | | OM-NOC as the control group | |
| --- | --- | --- | --- | --- |
|  | **OR(95%CI)** | ***P*** | **OR(95%CI)** | ***P*** |
| **First trimester^b^** |  |  |  |  |
| Sedentary |  |  |  |  |
| <10.5 | 1(Ref) | - | - | - |
| 10.6- | 1.29(0.45,10.74) | 0.33 | - | - |
| 13.5- | 1.27(0.56,6.00) | 0.25 | - | - |
| 18- | 3.25(2.05,5.17) | 0.001 | - | - |
| Housework |  |  |  |  |
| <39.75 | - | - | 1(Ref) | - |
| 39.8- | - | - | 0.84(0.44,1.63) | 0.61 |
| 44.3- | - | - | 0.48(0.26,0.89) | 0.02 |
| 54- | - | - | 0.38(0.20,0.61) | 0.02 |
| **Second trimester ^c^** |  |  |  |  |
| Occupation |  |  |  |  |
| <24 | 1(Ref) | - | 1(Ref) | - |
| 24- | 0.94(0.80,1.15) | 0.12 | 0.97(0.85,1.15) | 0.17 |
| 28.5- | 0.88(0.68,1.17) | 0.01 | 0.76(0.54,0.94) | 0.02 |
| 32.3- | 0.42(0.37,0.52) | 0.003 | 0.53(0.41,0.72) | 0.01 |
| Unhealthy dietary patterns |  |  |  |  |
| Q1 | 1(Ref) | - | 1(Ref) | - |
| Q2 | 2.72(1.77,4.20) | 0.43 | 1.81(1.00,3.24) | 0.05 |
| Q3 | 4.12(2.53,6.70) | <0.001 | 2.25(1.21,4.17) | 0.01 |
| Q4 | 7.43(4.22,10.64) | 0.001 | 7.44(3.11,12.45) | 0.01 |
| Sleep Quality Index (≥8) | 1.45(1.00,2.26) | 0.05 | 2.48(1.06,5.83) | 0.04 |
| **Third trimester^d^** |  |  |  |  |
| Depression | 2.11(1.40,3.21) | 0.002 | 3.21(1.45,4.97) | 0.01 |

a adjusted for paternal age, nationality, education level, BMI, and disease history.

b adjusted for statistically significant factors in paternal information and preparation for pregnancy；

c adjusted for statistically significant factors in paternal information , preparation for pregnancy and first trimester；

d adjusted for statistically significant factors in paternal information , preparation for pregnancy, first trimester and second trimester；

OM:mothers with overweight/obesity before pregnancy ;NOM:mothers without overweight/obesity before pregnancy ;OI:infants with overweight/obesity; NOI:infants without overweight/obesity.
