# Supplementary figures and images for: The risk prediction of intergenerational transmission of overweight and obesity between mothers and infants during pregnancy
Source: BMC Pregnancy Childbirth. 2024 Jan 23;24:74. doi: 10.1186/s12884-024-06268-7 (PMC10804797; doi:10.1186/s12884-024-06268-7)

Fig.S1 Flow chart of research object inclusion process

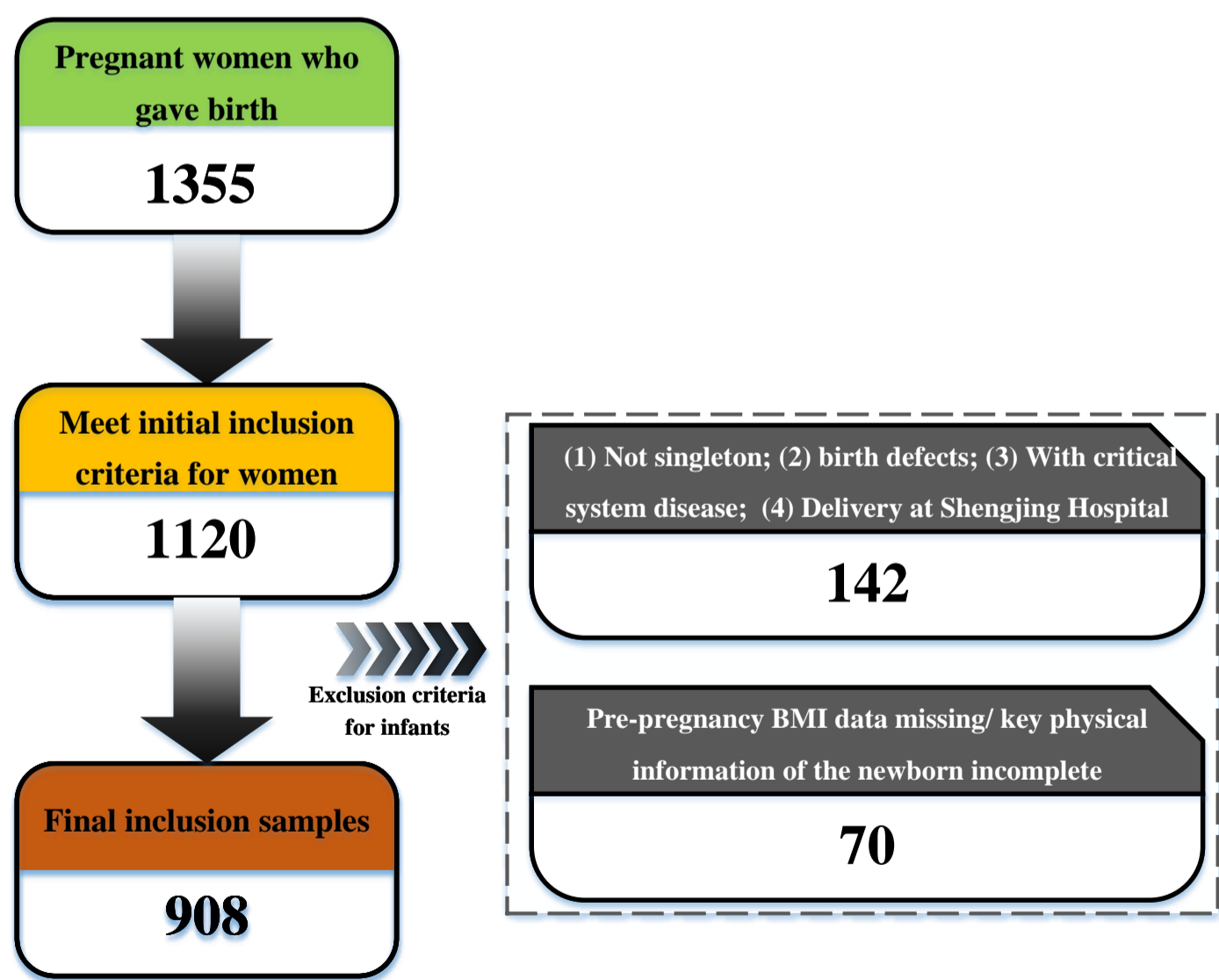

Supplement: Supplementary file 2 — Additional file 2. [file 12884_2024_6268_MOESM2_ESM.pdf]

Fig.S2 Flow chart for risk assessment

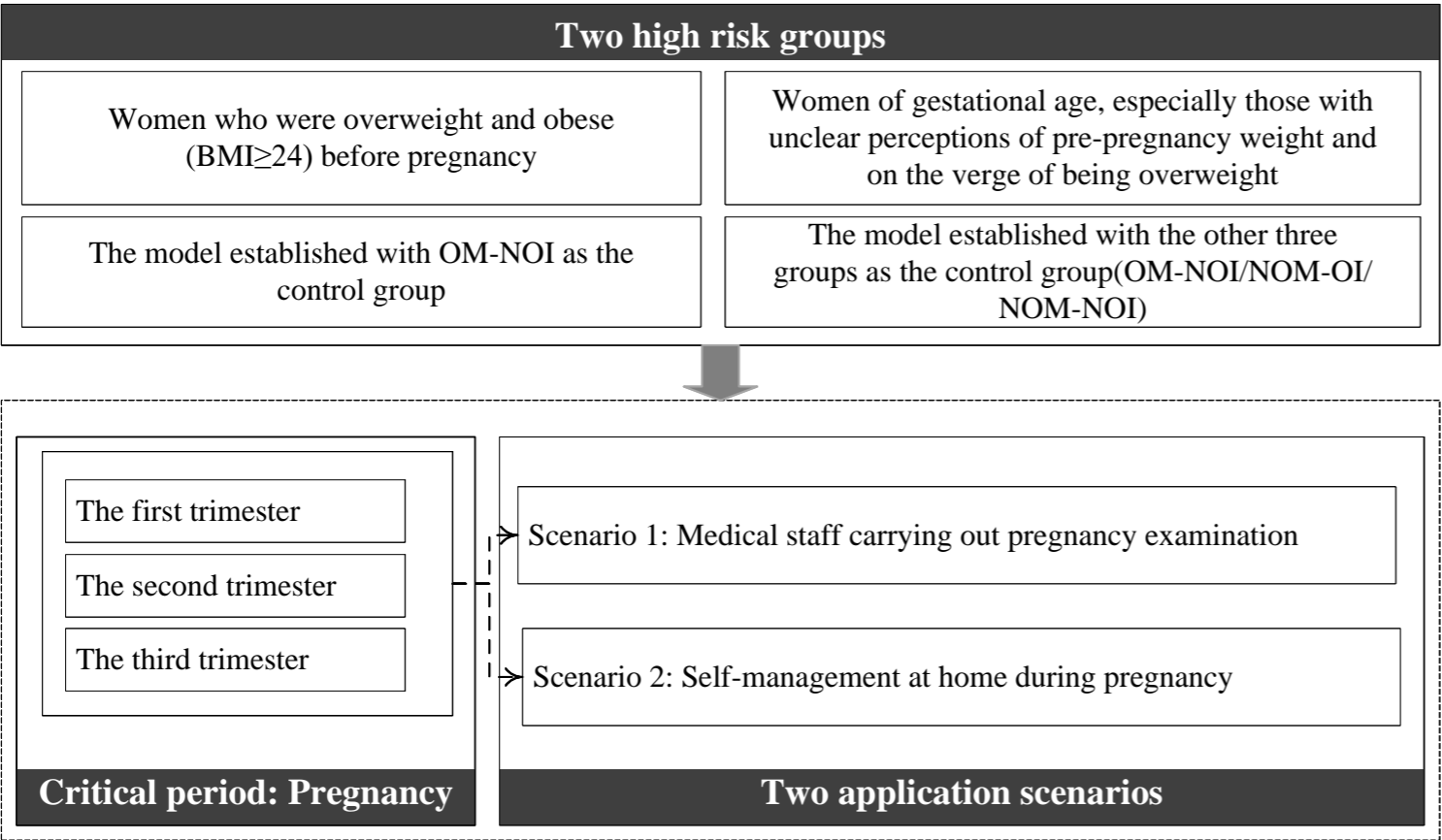

Supplement: Supplementary file 3 — Additional file 3. [file 12884_2024_6268_MOESM3_ESM.pdf]

Fig.S3 Investigation contents in each stage of the project

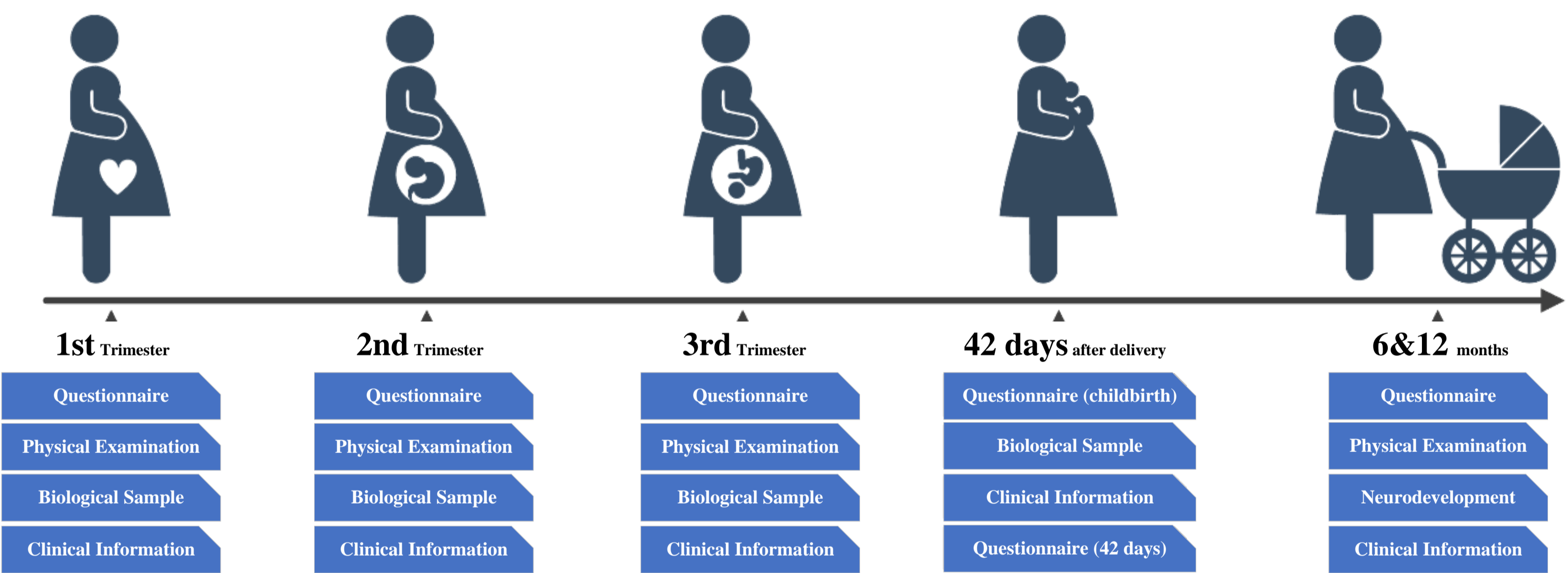

Supplement: Supplementary file 4 — Additional file 4. [file 12884_2024_6268_MOESM4_ESM.pdf]
